# Supplementary material for: Fixational drift is driven by diffusive dynamics in central neural circuitry
Source: Nat Commun. 2022 Mar 31;13:1697. doi: 10.1038/s41467-022-29201-y (PMC8971408; doi:10.1038/s41467-022-29201-y)
Supplement: Supplementary file 1 — Supplementary information file [file 41467_2022_29201_MOESM1_ESM.pdf]

Supplementary Information

Fixational drift is driven by central neural circuitry

Nadav Ben-Shushan, Nimrod Shaham, Mati Joshua, and Yoram Burak

Inventory of supporting information:

- Supplementary Notes
- 10 Extended data figures:
  1. **Correlation coefficient and χ sorted by their SE**
  2. **MSD curve: removal of measurement noise contribution**
  3. **The visual feedback**
  4. **Non-diffusive integrator effect on the measured statistics - simulations**
  5. **Fitting the MSD curve of monkey I**
  6. **Identifying OMNs vs. INNs**
  7. **Inter-saccade interval distribution**
  8. **Distribution of Spearman correlation coefficients**
  9. **Parameters of the computational model**
  10. **Spiking noise effect in the computational model**
- SI Data.1 – Parameters of the simulated oculomotor integrator network. The first column corresponds to the slopes of the neuronal tuning curves, $\boldsymbol{\zeta}$, the second to the offset at straight ahead gaze, $\boldsymbol{\lambda}^0$, and the third to the optimized values, $\boldsymbol{\eta}$.
- SI Data.2 – Parameters of the OMNs in simulations. The first column corresponds to the slopes of the neuronal tuning curves, k, the second to the eye velocity sensitivity, r, the third to the eye acceleration sensitivity, m, the fourth to the tuning curve threshold, E_T, and the fifth to the CV of each OMN.

Supplementary Notes

The explanatory power of a single OMN

In the main text we concluded that OMNs share a common input, since each OMN can explain a relatively large fraction of the variance in the measured eye position. Here we provide further details on the reasoning that leads to this conclusion.

First, consider a predictor of the eye motion, which is based on the activity of a single OMN. The coefficient of determination R^2 between the predictor and the actual motion is equal to the fraction of the variance in the motion that can be explained by the OMN activity. If the fixational eye motion is generated by noise in the OMN activity, and the noise is independent in different OMNs, then the variance in the eye motion is a sum over contributions from individual OMNs. Intuitively, the sum over explained variances cannot exceed the actual variance. More formally, it is straightforward to see that for a set of independent random variables $\{i\}$, whose coefficients of determination with some other variable are denoted by R_i^2, the sum $\sum_i R_i^2$ is bounded from above by unity (in our case, the independent random variables are the predictors of eye motion generated by each OMN, and the other variable is the actual motion of the eye). We conclude that if there are N_m OMNs that independently drive eye motion,

$$\left\langle R^2 \right\rangle \equiv \frac{\sum_i R_i^2}{N_m} \leq \frac{1}{N_m} \tag{S1}$$

where $\langle\rangle$ denotes averaging over the population of OMNs.

Under the assumption that the recorded OMNs are representative of the full population, we can attempt to estimate $\langle R^2 \rangle$ based on our data. In practice, we found that it was difficult to obtain a reliable estimate of R^2 directly from our data set, mostly due the large uncertaintly in the estimates of the covariances. We were able to obtain a more reliable estimate for the mean of $\langle R \rangle$, which could then be used to obtain a lower bound:

$$\langle R^2 \rangle \geq \langle R \rangle^2 \tag{S2}$$

Since N_m is estimated to be in the order of magnitude of a few thousands [1, 2], we would expect, based on Eqs. S1-S2 to obtain $\langle R \rangle^2 \lesssim 10^{-3}$, which is an order of magnitude smaller than our estimates. Thus, we can reject the hypothesis that the fixational eye motion originates from independent contributions of noise in the individual OMNs.

Contribution of oculomotor state to the CV of OMNs

We assessed the spiking variability of OMNs by examining the CV of the inter-spike interval during fixation. In doing so, we implicitly assumed that the firing rate remains fixed during each fixational

epoch over which the CV was measured. However, we have argued in this work that the firing rate varies during the fixational epoch, due to the diffusive dynamics within the oculomotor integrator. This raises a question, whether the diffusive dynamics within the oculomotor integrator affects our measurements of the CV. Here we show that the effect of the diffusion on the measurement of the CV can be safely neglected.

Let us assume that the instantaneous firing rate of a neuron is given by

$$R(t) = kE_{OI}(t) + R_0 \tag{S3}$$

which is a random variable due to the variability of E_{OI}, and that the CV is measured over an interval of duration Δt.

To estimate the CV of the ISI that arises from the diffusion in the oculommotor integrator, let us assume that there is no intrinsic spiking variability: hence, the inter-spike interval is given precisely by $1/R$. Assuming that the state of the integrator undergoes simple diffusion with diffusivity D, the variance of E_{OI} over the measurement interval is then given by

$$\begin{aligned} \langle\langle \Delta E_{OI}^2 \rangle_T\rangle &= \left\langle \frac{1}{T}\int_0^T \left[E_{OI}(t) - \langle E_{OI}\rangle_T\right]^2 \mathrm{dt} \right\rangle \\ &= 2D \cdot T/6 \end{aligned} \tag{S4}$$

where averaging is with respect to time $\langle \ldots \rangle_T$, and over instances of the random diffusive trajectory of E_{OI}. The CV of $1/R$ is then given by

$$\mathrm{CV}\left(\frac{1}{R}\right) = \frac{\mathrm{std}(1/R)}{\langle 1/R\rangle} \simeq \frac{\mathrm{std}(R)}{\langle R\rangle} \simeq \frac{k\sqrt{2D\cdot T}}{\sqrt{6}\,\langle R\rangle} \tag{S5}$$

In a typical fixation interval of 500 ms, with $k \sim 1/15$ spikes/s/arcmin, $2D \approx 100\,\mathrm{arcmin}^2/\mathrm{s}$ and $\langle R\rangle = 80$ Hz, we conclude that the CV of the inter-spike interval is given by

$$\mathrm{CV}\left(\frac{1}{R}\right) \approx 10^{-3} \tag{S6}$$

which is an order of magnitude smaller than the measured CV of OMNs. In addition note that under these biologically plausible assumptions $\sqrt{\langle \Delta R\rangle^2} \ll \langle R\rangle$. Hence, the approximations made in the above derivation are valid. We conclude that the contribution of the diffusive state of the integrator is negligible compared to other sources of noise in the OMNs. Similar argumentation holds for neurons within the oculomotor integrator.

Contributions of peripheral noise and feedback to the eye motion

Influence of spiking noise on the MSD curve

Our model includes spiking noise in both the integrator neurons and OMNs. The primary role of spiking noise is to drive diffusive dynamics along the continuous attractor of the OI. A secondary effect of spiking noise within the OI is to generate a noisy output to OMNs, which exists even if the internal state of the OI is fixed (i.e., in the absence of diffusion). Here we demonstrate that this latter source of variability, and the intrinsic spiking variability of the OMNs both have a negligible effect on the MSD curve over the experimentally relevant time scales. To show this we performed simulations in which the spiking noise in OMNs and in the output of the oculomotor integrator were eliminated by (1) simulating the dynamics of the oculmotor integrator with spiking neurons to drive diffusion, but using the instantaneous firing rates of oculomtor neurons (as determined by their synaptic input) instead of spikes as an input to the OMNs, and (2) using deterministic OMNs. Results are shown in Extended Data Fig.10 where we compare the full model against the model without the spiking noise. Note that relative to other sources of motion the spiking noise is consequential only at very short time scales $\sim 10^{-3} - 10^{-2}$ s. Beyond time lags of order 10^{-2} s the MSD curves with and without spiking noise are almost indistinguishable.

Visual feedback

As shown in the main text the diffusive dynamics within the oculomotor integrator, combined with the mechanic response properties of the ocular muscles and plant, is sufficient to explain the main qualitative features of the MSD curve: the superdiffusive nature of the motion up to time scales of at least several hundred ms, and the reduction in the logarithmic slope of the curve (to a value that still exceeds unity) beyond time scales that exceed approximately 100 ms. To obtain precise quantitative agreement between the MSD curve predicted by the model and the experimetntally measured curve, we found that it was necessary to include a mechanism that decreases the slope of the MSD curve at time scales exceeding 100ms, even beyond the decrease in the slope that was already predicted based on the other ingredients of the model.

We thus included in the model a simple yet biologically plausible visual feedback mechanism that suppresses motion of the target over the retina. The features of this feedback mechanism were motivated as follows. First, several studies have shown that the statistics of eye motion during fixational drift are influenced by the visual stimulus [3, 4, 5, 6, 7, 8, 9]. This indicates that fixational drift is influenced, at least to some extent, by the stimulus. Second, recent measurements of fixational motion in human subjects using optical eye trackers were shown to exhibit weak oscillatory behavior over long time scales, with a period of about 100-200 ms [10, 11]. These oscillatory features could be explained by the existence of a delayed visual feedback mechanism [10]. The oscillations identified in [10] are reminiscent of similar oscillations that were previously observed during smooth pursuit [12]. These observations have led to our simple model of the visual

feedback in which: (i) the brain generates an estimate of the recent drift velocity of the target on the retina by comparing a delayed estimate of the position of the target with the internal state of the OI, and (ii) this estimate, multiplied by a negative numerical prefactor, is fed as a velocity signal into the OI which drives motion in the opposite direction (Equations 29 and 19 in the main text).

Influence of the visual feedback on the MSD curve

The velocity feedback included in our model has several effects, illustrated in Extended Data Fig.4, which shows the MSD curve (Extended Data Fig.4a) and its logarithmic derivative (Extended Data Fig.4b) with and without visual feedback. For simplicity, and due to the very minor effect of OMN spiking noise on the MSD curves, we did not include spiking noise in these simulations. First, the visual feedback mechanism decreases the amplitude of the eye motion at all time lags (Extended Data Fig.4a). Second, it decreases the slope of the MSD curve (Extended Data Fig.4b). In terms of fitting the model to the data, the crucial effect of the feedback was the decrease in the slope of the MSD curve in comparison to the no-feedback case. This enables the model to fit well the MSD curve at both short and long time lags. Without the feedback we were only enable to fit well with the data in either short or long time lags (Extended Data Fig.5). In addition, the feedback decreases the amplitude of the MSD curve which facilitated the reach of a good match between the model and the data with biologically plausible choices for the parameters that control the magnitude of the diffusive motion, i.e. , with 15K neurons in the integrator with CV of ISI of 0.20, and 1K OMNs.

References

[1] McClung, J. R., Shall, M. S. & Goldberg, S. J. Motoneurons of the lateral and medial rectus extraocular muscles in squirrel monkey and cat. *Cells Tissues Organs* **168**, 220–227 (2001).

[2] Bach-y Rita, P. *The control of eye movements* (Elsevier, 2012).

[3] Nachmias, J. Determiners of the drift of the eye during monocular fixation. *JOSA* **51**, 761–766 (1961).

[4] Steinman, R. M., Haddad, G. M., Skavenski, A. A. & Wyman, D. Miniature eye movement. *Science* **181**, 810–819 (1973).

[5] Epelboim, J. & Kowler, E. Slow control with eccentric targets: evidence against a position-corrective model. *Vision research* **33**, 361–380 (1993).

[6] Poletti, M. & Rucci, M. Eye movements under various conditions of image fading. *Journal of vision* **10**, 6–6 (2010).

[7] Rucci, M. & Poletti, M. Control and functions of fixational eye movements. *Annual Review of Vision Science* **1**, 499–518 (2015).

[8] Intoy, J. & Rucci, M. Finely tuned eye movements enhance visual acuity. *Nature communications* **11**, 1–11 (2020).

[9] Malevich, T., Buonocore, A. & Hafed, Z. M. Rapid stimulus-driven modulation of slow ocular position drifts. *bioRxiv* (2020).

[10] Herrmann, C. J., Metzler, R. & Engbert, R. A self-avoiding walk with neural delays as a model of fixational eye movements. *Scientific reports* **7**, 1–17 (2017).

[11] Hohl, S. S. & Lisberger, S. G. Representation of perceptually invisible image motion in extrastriate visual area mt of macaque monkeys. *Journal of Neuroscience* **31**, 16561–16569 (2011).

[12] Goldreich, D., Krauzlis, R. & Lisberger, S. Effect of changing feedback delay on spontaneous oscillations in smooth pursuit eye movements of monkeys. *Journal of neurophysiology* **67**, 625–638 (1992).

[13] Joshua, M., Medina, J. F. & Lisberger, S. G. Diversity of neural responses in the brainstem during smooth pursuit eye movements constrains the circuit mechanisms of neural integration. *Journal of Neuroscience* **33**, 6633–6647 (2013).

[14] Sylvestre, P. A. & Cullen, K. E. Quantitative analysis of abducens neuron discharge dynamics during saccadic and slow eye movements. *Journal of neurophysiology* **82**, 2612–2632 (1999).

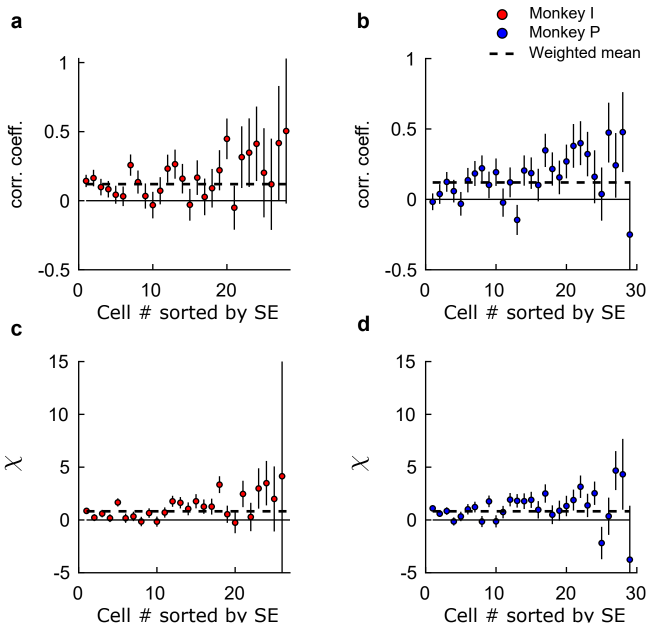

Extended Data Fig. 1: **Correlation coefficient and χ sorted by their SE a**, Correlation coefficients as measured for each OMN in monkey I (data are presented as mean values ± SEM). **b**, Same as in (a) but for monkey P. **c**, The estimated fraction of variance in the measured eye position which is due to the central source i.e. χ (see Methods) measured in monkey I. For clarity two cells with $\chi = 73 \pm 37,\ 44 \pm 41$ are not shown. (data are presented as mean values ± SEM). **d**, Same as in (c) but for monkey I. In all panels, cells are ordered by the standard error of the estimates. Distribution of fixational segments per cell is given in Extended Data Fig.7c.

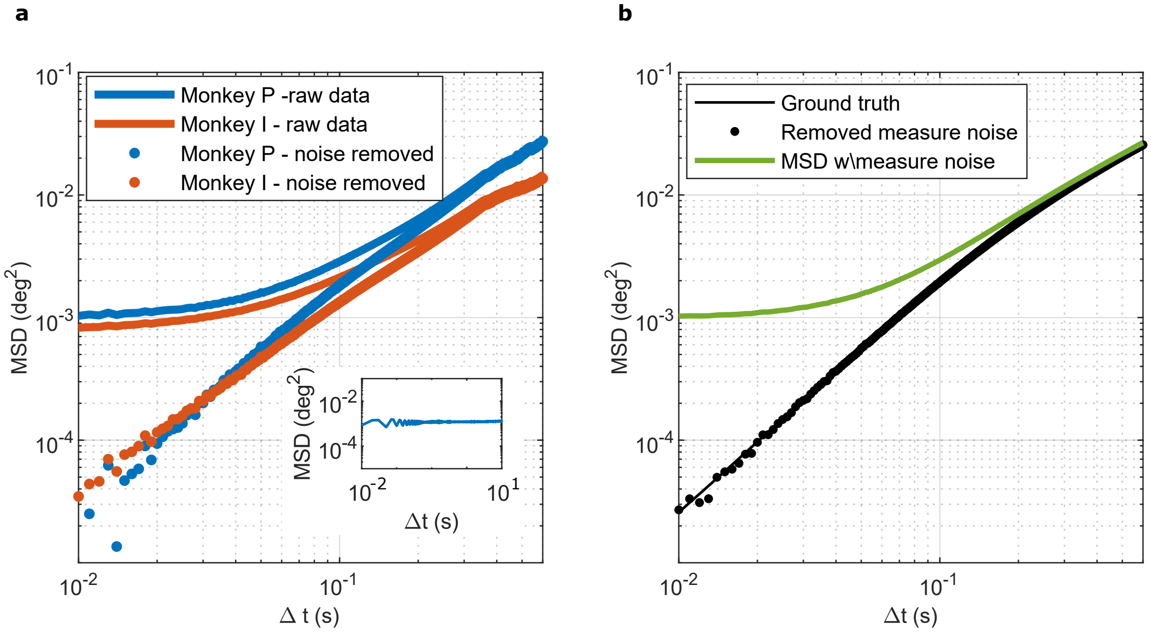

Extended Data Fig. 2: **MSD curve: removal of measurement noise contribution. a,** The measured MSD before and after removal of the contribution arising from measurement noise. Saturation of the MSD at short time lags is indicative of the existence of temporally uncorrelated measurement noise. The contribution of the noise to the MSD can be estimated as the value approached by the MSD at time lags close to zero, $\sim 10^{-3}$ deg^2. Adjusted MSD curves (colored circles) are obtained by subtracting this estimate of the measurement noise contribution from all data points. Inset: separate estimate for the measurement noise MSD, obtained by measuring the position of a coil fixed in space using a similar setup as in [13], confirm that the MSD arising from noise in the measurement apparatus is of similar order of magnitude as in our estimates. **b,** Validation of the noise removal methodology using our model. We simulated eye trajectories, to which we added Gaussian white noise representing the measurement noise. Solid green trace: MSD curve obtained from the noise contaminated eye trajectories. As the time lag approaches zero, the MSD saturates at a value equal to twice the variance of the Gaussian noise (10^{-3} deg^2 in the simulation, similar to the real data). Following the same protocol as in our analysis of the experimental data, we subtracted this estimate of the measurement noise from each point in the solid green MSD curve, resulting in an adjusted MSD curve (solid black circles). The solid black trace shows the MSD curve obtained from the simulated eye trajectories prior to noise addition, in excellent agreement with the MSD curve obtained by the noise removal methodology (solid black circles). Note the qualitative similarity to panel a.

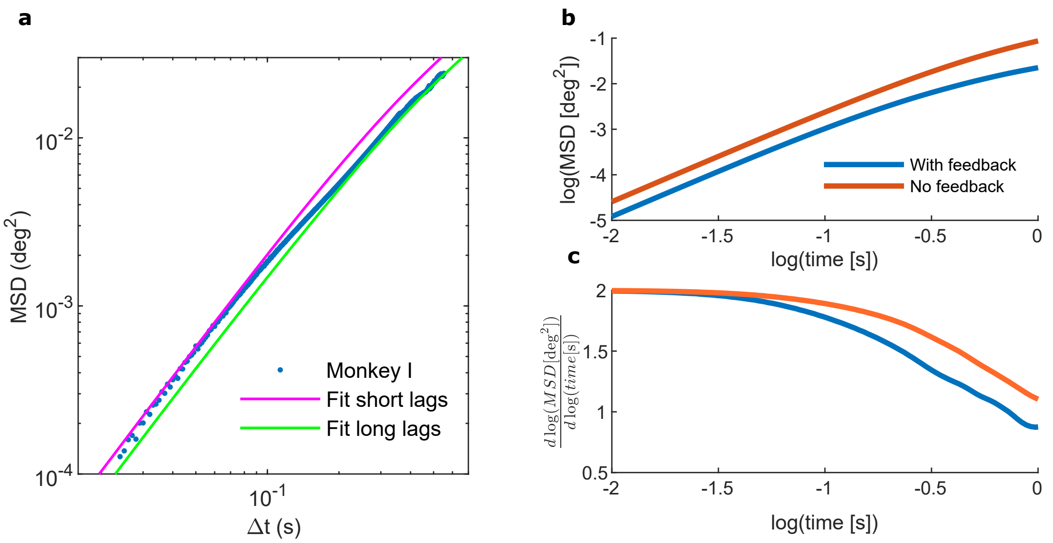

Extended Data Fig. 3: **The visual feedback.** **a**, Without the visual feedback mechanism, the model can not fit the measured data precisely: it either overestimates the MSD at large time lags or underestimates the MSD at short time lags. Fitting to the behavior at short time scales (magenta) was obtained with $N = 40,000$ oculomotor integrator neurons of CV$\sim$ 0.23, and $N_m = 1,200$ OMNs. Fitting the behavior at long time scales (green) was obtained with $N = 40,000$ oculomotor integrator neurons with CV$\sim$ 0.20, and $N_m = 1,500$ OMNs. **b**, Feedback decreases the amplitude of the MSD at all time scales, but more significantly at $\Delta t \gtrsim \tau_f$. **c**, Feedback decreases the logarithmic slope of the MSD curve at $\Delta t \gtrsim 100$ ms.

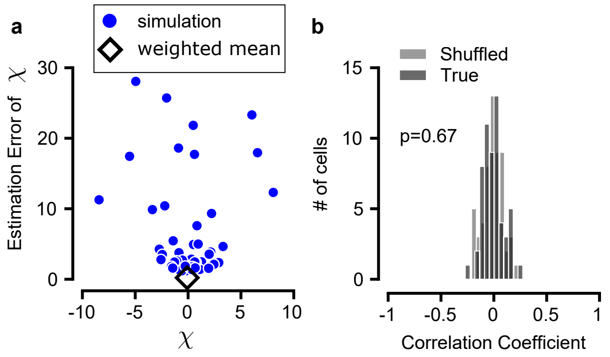

Extended Data Fig. 4: **Non-diffusive integrator effect on the measured statistics - simulations.** Analysis of simulated data (spiking activity and eye trajectories) generated by an alternative model, in which there is no central contribution to the eye motion. OMNs receive constant input corresponding to straight gaze, and eye motion is driven solely by the spiking variability of the OMNs. The number of OMNs and trials per OMN is as in Fig. **??**e, and similar to the analysis of the experimental data. **a,** Estimates of χ and their estimation errors. Estimation errors for individual OMNs and the spread of the estimates are large, similar to Fig. **??**e. However, the weighted average of χ obtained from all the OMNs, $\langle \chi \rangle = -0.09 \pm 0.22$ indicates that there is no dominant central contribution to the motion, and is in agreement with the correct value $\chi = 0$. **b,** Distribution of correlation coefficients between eye displacement over 350 ms intervals, and their estimates based on the spikes of individual OMNs (similar to Figs. **??**d and **??**e). The distribution is centered around zero, $\langle R \rangle = -0.006 \pm 0.013$, and is compatible with the distribution obtained after shuffling (gray, $p = 0.67$), as expected since the contribution of individual OMN spikes to the variance of the motion is very small, of order $1/N_m$.

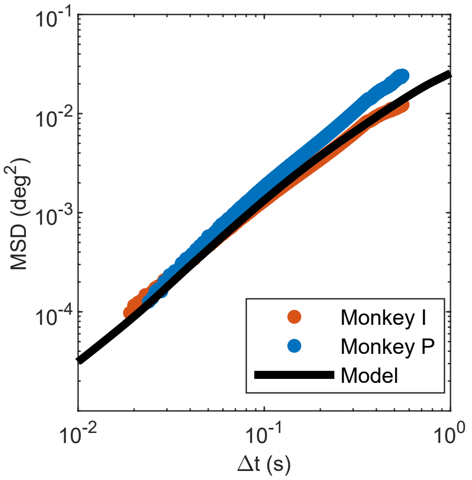

Extended Data Fig. 5: **Fitting the MSD curve of monkey I.** The MSD curve of monkey I can be fit to the model with minor modifications to the parameters from the values used in Fig.5d: (1) The number of OMNs was decreased from 1,000 to 900. (2) The CV of OMNs was determined from the linear relation $\text{CV} = 10^{-2} \times [8.0 + (0.18\ \text{s}^{-1}) \times \text{ISI}]$. (3) The feedback strength parameter A in equation (29) was increased from 0.015 to 0.060.

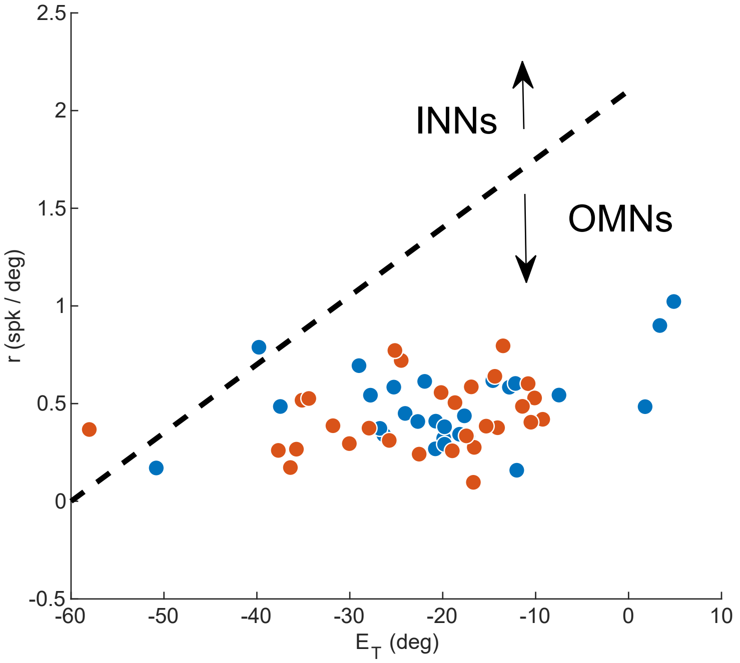

Extended Data Fig. 6: **Identifying OMNs vs. INNs.** Recorded cells in our data set were identified as OMNs vs. internuclear neurons (INNs) using a criterion based on tuning curve parameters, as in [14] (dashed black trace): the eye position threshold E_T and the eye velocity sensitivity r. (Two cells which were close to the separating criteria defined in [14] from above were included in the analysis. The inclusion of these cells did not qualitatively affect any of the results.)

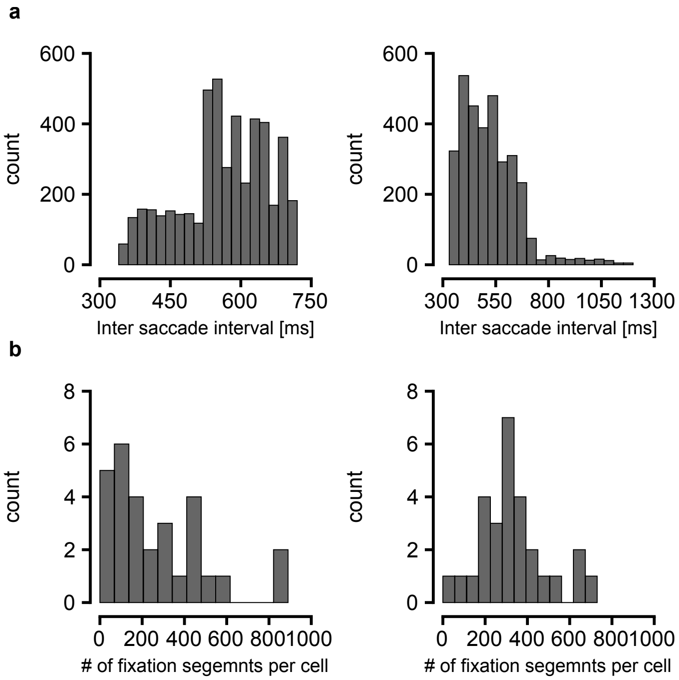

Extended Data Fig. 7: **Statistics of fixational segments extracted from the recorded eye trajectories.** **a** Durations of the analyzed inter-saccade intervals (Left: distribution in Monkey I, right: distribution in Monkey P). **b** Number of inter-saccade intervals of 350 ms length for monkey I (left) and monkey P (right).

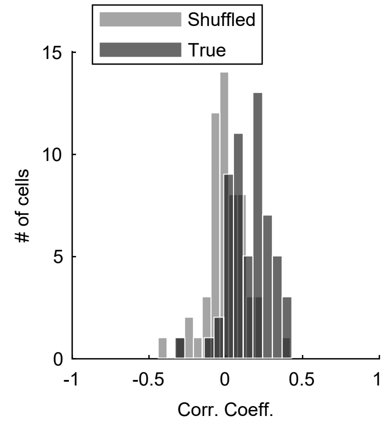

Extended Data Fig. 8: **Distribution of Spearman correlation coefficients.** The non-parametric correlation coefficient between δE and $\delta\hat{E}$ demonstrates a positive and significant mean value of 0.16 with one sided t-test p-value 3×10^{-12} (compare with Fig.2d).

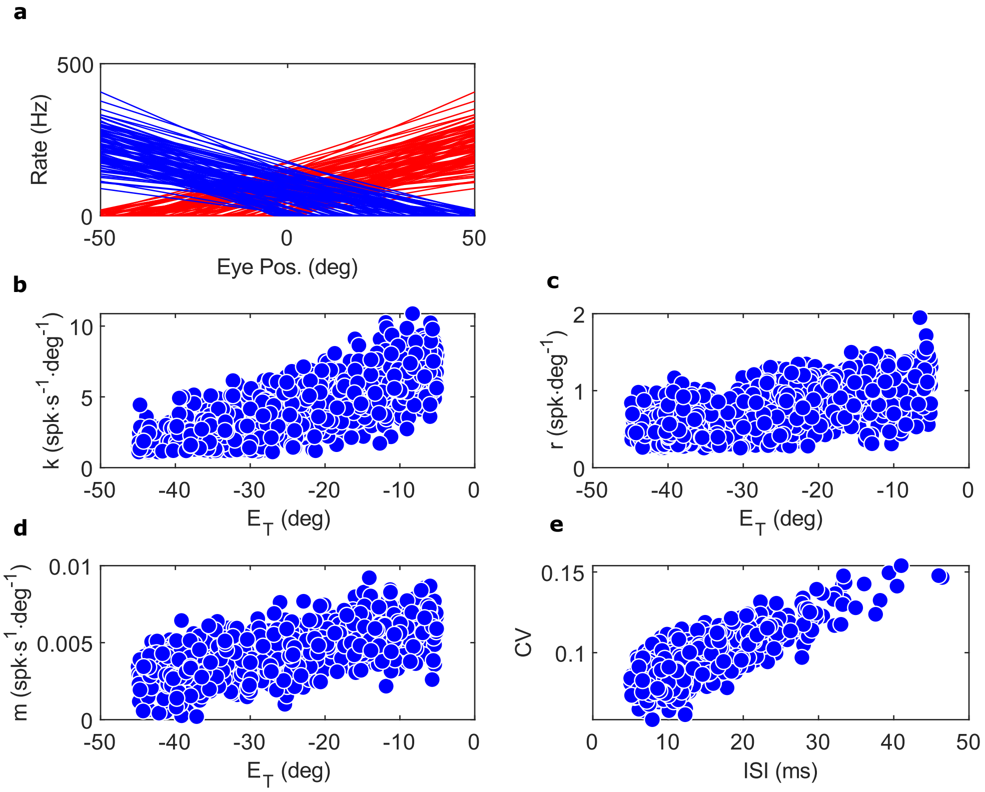

Extended Data Fig. 9: **Parameters of the computational model.** Visualization of the parameters used in the computational model. **a**, Tuning curves of neurons in the OI, red (blue) color corresponds to left (right) populations. For clarity only 100 representative tuning curves are shown from each population. **b,c,d**, The parameters which define the OMNs response as defined in equation(1). **e**, The CV of each OMN at straight ahead gaze.

.

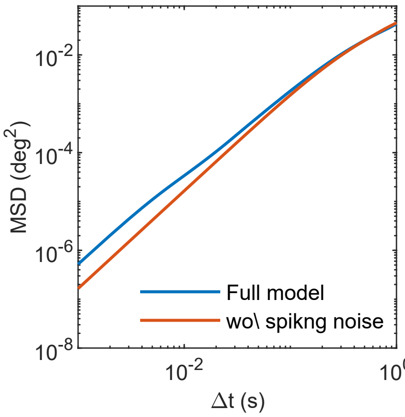

Extended Data Fig. 10: **Spiking noise effect in the computational model.** MSD curves generated by the full model, and by a model which excludes spiking noise, both in the output of the oculomotor integrator and in the OMNs (stochastic spiking was still included in the internal dynamics of the oculomotor integrator to drive diffusion). The spiking noise effect is negligible at time lags > 50ms, and contributes significantly to the MSD at smaller time lags, up to about half of the variance at a time lag of 1ms.
